# Treatment of Aspergillosis

**DOI:** 10.3390/jof4030098

**Published:** 2018-08-19

**Authors:** Jeffrey D. Jenks, Martin Hoenigl

**Affiliations:** 1 Department of Medicine, University of California–San Diego, San Diego, CA 92103, USA; jjenks@ucsd.edu; 2 Division of Infectious Diseases, Department of Medicine, University of California–San Diego, San Diego, CA 92103, USA; 3 Section of Infectious Diseases and Tropical Medicine and Division of Pulmonology, Medical University of Graz, Graz 8036, Austria

**Keywords:** *Aspergillus*, posaconazole, amphotericin, isavuconazole, voriconazole, itraconzole, invasive aspergillosis, chronic pulmonary aspergillosis, diagnosis, interleukin 8

## Abstract

Infections caused by *Aspergillus* spp. remain associated with high morbidity and mortality. While mold-active antifungal prophylaxis has led to a decrease of occurrence of invasive aspergillosis (IA) in those patients most at risk for infection, breakthrough IA does occur and remains difficult to diagnose due to low sensitivities of mycological tests for IA. IA is also increasingly observed in other non-neutropenic patient groups, where clinical presentation is atypical and diagnosis remains challenging. Early and targeted systemic antifungal treatment remains the most important predictive factor for a successful outcome in immunocompromised individuals. Recent guidelines recommend voriconazole and/or isavuconazole for the primary treatment of IA, with liposomal amphotericin B being the first alternative, and posaconazole, as well as echinocandins, primarily recommended for salvage treatment. Few studies have evaluated treatment options for chronic pulmonary aspergillosis (CPA), where long-term oral itraconazole or voriconazole remain the treatment of choice.

## 1. Introduction

*Aspergillus* spp. are filamentous, environmental fungi that cause a wide spectrum of infections in humans, including hypersensitivity reactions, chronic pulmonary infections, and acute life-threatening infections, the latter occurring primarily in immunocompromised individuals [1,2,3,4]. Of over 250 species of *Aspergillus*, fewer than 40 are known to cause infections in humans [5], and of these, *Aspergillus fumigatus* is the most common cause of infections in humans and is the most common cause of serious, invasive disease. 

The 2017 global estimates indicated that more than 3,000,000 cases of chronic pulmonary aspergillosis (CPA), and ≈250,000 cases of invasive aspergillosis (IA) occur annually [6]. Prior population-based estimates of invasive aspergillosis (IA) in the 1990s suggest an annual incidence rate of 1 to 2 cases of aspergillosis per 100,000 population [7], although given the increasing number of solid organ transplant (SOT) and stem cell transplant (SCT) recipients, as well as the increased use of immunosuppression, this study likely underestimates the current incidence of IA. In high-risk populations, such as SCT recipients, the 12-month incidence of invasive fungal infections (IFIs) is 3.4%, with IA causing around half of those infections [3]. In SOT recipients, the annual incidence of IA was 0.65% in one large study [8]. 

IA most commonly involves the lungs, where individuals present with signs and symptoms of pneumonia including fever, chest pain, and hemoptysis, although other organ systems may be involved. Diagnostic modalities are evolving, but *Aspergillus* infections are most commonly diagnosed by culture, histopathology, or by galactomannan antigen detection in blood or bronchoalveolar lavage fluid (BALF). The optimal management of infections from IA includes prevention, early diagnosis, early initiation of antifungal therapy, reduction in immunosuppressive therapy if possible, and in some cases, surgery. Amphotericin B was an early treatment option for IA and its newer lipid-based formulations are still used in some instances, although newer options including voriconazole and isavuconazole are now often preferred.

CPA is progressing more slowly than IA and affects patients with pre-existing pulmonary pathology, such as active or previous tuberculosis and non-tuberculosis mycobacterial infection, previous surgery for lung cancer, chronic obstructive pulmonary disease (COPD), or sarcoidosis, although the later may also be at risk for IA due to concomitant immunosuppressive treatment [1,9]. 

## 2. Clinical Manifestations and Diagnosis 

IA is primarily found in the lungs but may disseminate to other organs. Signs and symptoms depend on which organs are affected, and may include cough, hemoptysis, dyspnea, chest pain, skin lesions, or neurological symptoms. Importantly, pathogenesis of IA and associated signs and symptoms, as well as radiological presentation and diagnostic test performance, differs markedly between patients with neutropenia (i.e., primarily angioinvasive growth) and non-neutropenic patients (i.e., primarily airway invasive growth) [10,11]. While fever is present in >95% of neutropenic patients with IA, it is present in only 50–70% of non-neutropenic patients with IA [12], and cough and chest pain may be less frequently observed in non-neutropenic patients [12]. Radiological presentation in chest computed tomography (CT) differs by neutrophil count, with “typical” signs of IA including the halo sign or air crescent sign being frequently observed in neutropenic patients, while non-specific infiltrates or consolidations are most frequently observed in non-neutropenic patients [13,14]. Strikingly, “typical” radiologic signs, as defined by consensus definitions of IA, are present in only 30–50% of non-neutropenic proven IA cases [13]. As a consequence, IA remains difficult to diagnose, particularly in non-neutropenic patients where clinical presentation and radiological signs are mostly atypical. Therefore, diagnosis of IA is often delayed, and IA remains associated with high mortality rates of around 30–40% [15].

Due to the absence of a single “gold standard” test for the diagnosis of IA, different mycological assays are employed and combined with clinical, radiological, and histological methods. Mycological diagnostic approaches for IA include fungal culture from BALF and biopsies; immunodetection of the cell wall component galactomannan (GM) in serum, BALF, and urine; detection of the cell wall component 1,3-β-d-glucan (BDG) via factor G activation in serum; detection of *Aspergillus*-specific siderophores in BALF or urine; detection of an *Aspergillus*-specific cell wall protein via a lateral flow device test (LFD); and detection of *Aspergillus*-specific DNA via PCR in blood and BALF [16,17,18,19,20,21,22,23,24,25,26,27,28,29,30,31,32,33]. The most commonly used non-culture-based approach for IA diagnosis is GM detection in serum and BALF. In contrast to BALF GM detection, performance of serum GM is heavily influenced by neutrophil status of the host with sensitivities over 60–70% in neutropenic patients with angioinvasive growth but sensitivities are often <20% in non-neutropenic patients with airway invasive growth [34,35,36]. Another factor that reduces sensitivities of all diagnostic tests for IA, including culture, GM, PCR, and LFD is ongoing mold-active antifungal prophylaxis or treatment [37,38,39,40]. Given the reduced sensitivities of all available diagnostic tests in patients receiving mold-active antifungals, combination of multiple diagnostic tests and biomarkers from BALF and blood is the currently recommended approach, and has been shown to be associated with a significant increase of sensitivities, while specificities were only reduced slightly [19,20,23,30,32,41,42,43]. The most promising combinations include PCR and/or the *Aspergillus*-specific LFD and/or GM from BALF with serum GM and/or very high levels of serum interleukin 8, which has recently been shown to be highly sensitive and specific when combined with BALF LFD or PCR [19,22,23,26,27,30,32,33].

CPA is characterized by slowly progressive destruction of lung parenchyma, in the form of single of multiple cavities, nodules, infiltrates, or fibrosis, with or without an aspergilloma [2]. Due to the nonspecific and indolent presentation, diagnosis may be delayed or missed, resulting in severe morbidity. Diagnostic criteria include presence of respiratory and/or constitutional symptoms for at least 3 months, suggestive abnormalities on imaging, and serological or microbiological evidence of *Aspergillus* [44]. For mycological evidence, *Aspergillus* specific IgG has a central role, while biomarkers and tests for invasive growth, including GM, are mostly negative [44,45].

## 3. Treatment of *Aspergillus* Infections

### 3.1. Treatment of Invasive Aspergillosis

Early systemic antifungal treatment is of primary importance for survival against IA, while surgery plays an important role mainly in rarer disease manifestations of invasive disease including, e.g., sinusitis and osteomyelitis. Pharmacokinetic characteristics of broad-spectrum triazoles, liposomal amphotericin B, and echinocandins are reviewed in Table 1. The indications for surgery for IA have been extensively reviewed before [46]. The same applies to CNS infections and endophthalmitis where drug penetration considerations are particularly important (see also Table 1) [47].

#### 3.1.1. Amphotericin B

Intravenous (IV) amphotericin B, a polyene, binds to ergosterol in fungal membranes forming pores and leading to cell death. It is used to treat a number of fungal infections, including IA, mucormycosis, cryptococcal meningitis, and invasive candidiasis. Investigation of liposomal amphotericin B (L-AmB) has shown a positive correlation between in-vitro susceptibility testing and positive clinical outcomes in patients with IA. In a study of 29 patients with IA following SCT, all six patients with a mean inhibitory concentration (MIC) of <2 mg/L to L-AmB survived, while 22/23 patients with isolates with an MIC ≥2 mg/L died [53]. Another study using a pharmacokinetic-pharmacodynamic (PK-PD) model simulating free amphotericin B serum concentrations found the estimated breakpoints for susceptibility, intermediate susceptibility, and resistance for *A. fumigatus* to be ≤0.5, 1, and ≥2, respectively [54]. Overall, L-AmB remains sensitive against most *Aspergillus* spp., although increasing rates of elevated MICs >2 have recently been reported for *Aspergillus fumigatus* (>40% of isolates MICs >2), and *Apergillus flavus* (>80% of isolates) [55]. *Aspergillus terreus* shows intrinsic resistance [56].

In a prospective, randomized, multicenter trial of patients with either hematologic malignancy or status post SCT, patients were given either L-AmB at 1 mg/kg or L-AmB at 4 mg/kg daily. Of 87 evaluable patients, there was no difference in radiologic response, 6-month survival rates, or deaths between the two groups. The six-month survival rate was around 40% in both groups, with about 50% of those deaths thought to be related to IA [57]. Another multi-center randomized trial investigated L-AmB at either 3 mg/kd/day or 10 mg/kg/day for 14 days followed by 3 mg/kg per day. The vast majority of patients in this study had underlying hematologic malignancy and IA accounted for 97% of invasive fungal infections. There was no difference in clinical response or survival between the two groups, with clinical response in 50% of patients and 12-week survival of 72% in the 3 mg/kg per day cohort [58]. In a pooled analysis of published trials investigating L-AmB for the treatment of IA and other filamentous fungal infections in immunocompromised individuals, favorable response rates were observed in about 51% of cases of probable or proven invasive fungal infections [59]. While L-AmB remains an important agent for treatment of IA and other invasive mold infections [60], high rates of toxicity and side effects from L-AmB, including infusion-related reactions, phlebitis during infusion, gastrointestinal side effects such as diarrhea, nausea, and vomiting, transaminitis, electrolyte derangements, and nephrotoxicity have led to the investigation of other, better tolerated agents [61].

#### 3.1.2. Azoles

Broad-spectrum triazoles inhibit ergosterol synthesis in the membrane sterol of fungi leading to cell death. Several of the newer-generation triazoles, particularly voriconazole and isavuconazole, are appealing alternatives to the use of L-AmB. While azole resistant clinical *Aspergillus* isolates have been reported from many parts of the world, resistance rates remain low in most settings, not influencing primary choice of antifungal therapy [62]. Pharmacokinetics of a broad spectrum differ significantly, and intra- and interpatient variability of plasma levels, but also variability in intracellular concentrations remains an issue [63]. Therapeutic drug monitoring (TDM) may be required to achieve efficacy and also to avoid toxicity in particular for voriconazole in treatment and prophylactic settings [64,65,66], but also for posaconazole oral suspension (efficacy only) [67,68,69], which is associated with significantly lower posaconazole plasma concentrations when compared to tablet or IV formulations [70], while there are currently no recommendations of TDM for isavuconazole [63,71].

Voriconazole, available in tablet, IV, and suspension formulation, is approved for the treatment of IA, *Candida* infections, scedosporiosis, and furasiosis. A prospective randomized trial in patients with underlying hematologic disease and IA investigated 144 patients randomized to receive IV voriconazole and 133 patients randomized to receive IV amphotericin B deoxycholate. The primary outcome was partial or complete response to therapy. At twelve weeks, 52.8% of patients met the primary outcome in the voriconazole group (a complete response of 20.8% and a partial response of 31.9%) versus 31.6% in the amphotericin group (a complete response of 16.5% and a partial response of 15.0%), a statistically significant difference. At 12 weeks, the survival rate was 70.8% in the voriconazole group versus 57.9% in the amphotericin group, which again was statistically significant. In addition, there were fewer drug-related adverse events in the voriconazole group, although side effects to treatment, such as blurry vision and photophobia, were common [72] 

Isavuconazole, available in capsule and IV formulation, is another triazole preferred for the treatment of IA [63,73,74,75]. In the large randomized SECURE trial, 527 patients with invasive mold infection were randomized to receive either IV isavuconazole followed by IV or oral (PO) isavuconazole versus IV voriconazole followed by IV or PO voriconazole. The majority of patients in both groups had underlying hematologic malignancy (82% in the isavuconazole group versus 86% in the voriconazole group). The primary endpoint was all-cause mortality at 6 weeks and *Aspergillus* spp were the cause of infection in 30% of patients in both groups. At 6 weeks, 19% of patients in the isavuconazole group died compared to 20% in the voriconazole group, a difference that did not meet statistical significance. Drug-related adverse events were significantly higher in the voriconazole group compared to the isavuconazole group (60% versus 42%, *p* < 0.001), and permanent drug discontinuation was lower in the isavuconazole group compared to the voriconazole group (8% versus 14%) [76].

A number of studies have suggested that posaconazole, available in delayed-release tablets, oral suspension, and IV formulation, may be an option to treat refractory IA [77]. An open-labeled trial investigated the use of posaconazole as a salvage therapy for the treatment of IA in patients that were refractory or intolerant to other treatment, of which 80% of patients had underlying hematologic malignancy. Of 107 patients who received posaconazole oral suspension, 42% had a treatment response compared to 26% of the 86 external control patients (*p* = 0.006) [78]. In another prospective trial of patients with refractory IA, 53 patients received posaconazole given orally or via an enteral feeding tube, 52 received lipid formulation of amphotericin B (LPD/AMB), and 38 patients received a combination of caspofungin plus LPD/AMB. The primary outcome was clinical improvement or resolution at 12 weeks and over 90% of patients had underlying hematologic malignancy. The primary outcome was met in 40% of patients in the posaconazole group, 8% in the LPD/AMB group, and 11% in the combination group (*p* < 0.002). At 12 months, 40% of patients died in the posaconazole group compared to 65% in the LPD/AMB group and 68% in the combination group (*p* < 0.008) [79]. 

#### 3.1.3. Echinocandins

The echinocandins, which inhibit β-1,3-glucan synthesis in the cell wall of fungi, have also been investigated for the treatment of IA. In an open-labeled, non-randomized, single-arm study, patients with IA following SCT were given at least 15 days of IV caspofungin (a median duration of 24 days). The primary endpoint was partial or complete clinical response. Enrollment was stopped early due to slow enrollment and the study was under-powered. At 12 weeks, 8 patients (33%) had complete or partial response to therapy and survival at 6 and 12 weeks was 79% and 50%, respectively [80]. In another open-label, non-randomized, single-arm study, patients with underlying hematologic malignancy or following SCT were given at least 15 days of caspofungin (a median duration of 27 days). The primary endpoint was partial or complete clinical response. At the end of treatment 1 patient (1/61) had a complete response to treatment and 19 patients (19/61) had a partial response. At 6 and 12 weeks the survival rate was 66% and 53%, respectively. Patients were not followed beyond 12 weeks [81]. Lastly, another study looked at caspofungin for the treatment of refractory IA and compared treatment responses to a historical cohort. The majority of patients had underlying hematologic disease. Of 83 patients who received caspofungin, 45% experienced a complete or partial clinical response to treatment, compared to a 16% response rate in the historical cohort, of which the majority received amphotericin B with or without itraconazole [82].

#### 3.1.4. Combination Therapy

In a large randomized, double-blind, placebo-controlled multicenter trial, 454 patients with hematological malignancies and suspected or documented IA were randomly assigned to primary treatment with voriconazole and anidulafungin or voriconazole and placebo. Primary analysis was done in the modified intention-to-treat population of 277 patients in whom IA was confirmed. Mortality rates at 6 weeks were 19.3% for combination therapy and 27.5% for monotherapy (difference, −8.2 percentage points [95% CI, −19.0 to 1.5]; *p* = 0.087). In a post hoc analysis of 218 patients who had IA diagnosis established by radiographic findings and galactomannan positivity, 6-week mortality was lower in combination therapy than monotherapy (15.7% vs. 27.3%; difference, −11.5 percentage points [CI, −22.7 to −0.4]; *p* = 0.037) [83]. While the study was insufficiently powered to detect a difference in the main analysis, results indicate that combination therapy may be associated with better survival [83]. 

A multinational, open-label, non-comparative study investigated IV micafungin, alone or in combination with other agents, for the treatment of IA. The primary outcome was partial or complete clinical response. Of 225 patients who met inclusion criteria, the majority were SCT recipients or had an underlying hematologic malignancy. Of those who received micafungin as primary therapy, 17/29 received combination therapy, with 2/17 experiencing a complete response to treatment and 3/17 a partial response (total response of 29%); 12/29 received monotherapy with micafungin with 0% experiencing a complete response and 6/12 experienced a partial response (a total response of 50%). Of those who received micafungin as salvage therapy, 174/196 received combination therapy with 13/174 experiencing a complete response and 47/174 a partial response (a total response of 34.5%); 22/196 received micafungin monotherapy with 3/22 experiencing a complete response and 6/22 a partial response (a total response of 41%) [84]. An open-label, non-comparative study of SCT recipients investigated micafungin alone or in combination with other agents for the treatment of IA. Most patients (89%) had refractory IA that did not respond to initial first-line therapy. The primary endpoint was response to therapy. Of 98 patients, 8 (8%) received micafungin monotherapy with a treatment response rate of 38% (3/8). Of 90 patients (92%) who were treated with combination therapy, the treatment response rate was 24% (22/90), with all but one of those treatment responses being in patients with refractory IA [85].

Another study investigated caspofungin in combination with other agents for the treatment of refractory IA. Of 53 patients, 30% (16/53) received caspofungin plus amphotericin B and 70% (37/53) received caspofungin plus a triazole. Partial or complete resolution at the end of therapy and at Day 84 was 55% (29/53) and 49% (25/51), respectively. Survival at Day 84 was 55% [86]. Lastly, another study looked at voriconazole plus caspofungin for the primary treatment of IA in SOT recipients and compared outcomes to a historical cohort of SCT recipients who received LPD/AMB. At 90 days, survival was 67.5% (27/40) in the combination therapy group versus 51% (24/47) in the historical control group (*p* = 0.117) [87]. 

#### 3.1.5. Guideline Recommendations

Current recommendations are summarized in Table 2. The Infectious Diseases Society of America (IDSA) 2016 guidelines recommend voriconazole as first-line treatment for IA (AI recommendation) with liposomal amphotericin B and isavuconazole as alternative options (AII recommendation). L-AmB is an option for salvage therapy (AII recommendation) [88]. The more recently updated guidelines of the European Society of Clinical Microbiology and Infectious Diseases (ESCMID) and the European Confederation of Medical Mycology (ECMM), as well as the guidelines of the European Conference on Infections in Leukemia (ECIL-6), both recommend voriconazole and isavuconazole as the preferred agents for the treatment of IA in patients with underlying hematologic malignancy (AI—AII recommendation and A1 recommendation, respectively). The ESCMID/ECMM 2017 and ECIL-6 recommend L-AmB as a second-line option (BII and BI recommendation, respectively) 1]. While the IDSA does not recommend primary therapy with an echinocandin (AII), ESCMID/ECMM and ECIL-6 only marginally recommend primary treatment with an echinocandin (CII recommendations).

The IDSA gives combination treatment with voriconazole and an echinocandin a weak recommendation, offering consideration for this approach in select patients (CII recommendation). In line, ESCMID/ECMM 2017 and ECIL-6 both only marginally recommend combination antifungal therapy with voriconazole plus an echinocandin for the primary treatment of IA (CI recommendation). Posaconazole is given a BII grade by both the ESCMID/ECMM 2017 and ECIL-6 and is recommended by the IDSA for use as a salvage therapy for IA (AII recommendation). In addition, the ESCMID/ECMM recommends voriconazole as a first-line treatment of IA in patients without hematologic malignancies (AIII recommendation). The IDSA recommends that antifungal therapy be continued for a minimum of 6–12 weeks, with duration based on factors such as severity of infection, duration of immunosuppression, and response to therapy. The ESCMID/ECMM recommends treatment duration be based primarily on treatment response and immune reconstitution.

While currently available guidelines are targeted towards a small number of high-resource countries, they mostly fail to provide guidance for low- and middle-income countries (LMICs). This will be changed by the “One World–One Guideline” initiative, which has been spearheaded by the European Confederation of Medical Mycology and has the goal to unify the medical mycology community, by creating guidelines that are applicable worldwide including low- and middle-income countries [71].

#### 3.1.6. Azole Resistance in *Aspergillus Fumigatus*

Although voriconazole and isavuconazole are the first-line agents for the treatment of IA, resistance to these agents is emerging [89]. A single-center study in Brazil investigated susceptibility profiles for *Aspergillus* isolates, of which *A. fumigatus* represented 74% of the 228 isolates. Nine patients showed high MIC values to at least one azole, although none showed complete resistance. Of additional concern, 43% of *A. fumigatus* isolates had an MIC >2 mg/L to amphotericin B, an important option for treatment of refractory IA [56]. In a 2016 nationwide survey of Dutch Hematology Centers, of 784 patients in which *A. fumigatus* was isolated, 101 isolates (12.9%) showed resistance to triazoles, an increase from 10.7% of isolates in 2015 and 7.2% of isolates in 2014. Among individual Hematology Centers, resistance ranged from 9.5% to 20.5%. This increasing resistance to triazoles is thought to be due to the environmental use of azole fungicides. Because of high local resistance to triazoles in one Dutch Hematology Center, L-AmB is frequently used as first-line therapy for IA [90]. Another Dutch cohort study found that triazole resistance was found in 23/144 (16%) of patients with influenza-associated IA, and that the detection of resistance to voriconazole was highly associated with treatment failure [91]. Thus, there is a clear need for continued vigilance for triazole-resistant strains of *A. fumigatus*. 

#### 3.1.7. New Agents to Treat Invasive Aspergillosis 

While there has been substantial improvement in treatment of IA, associated with decreasing mortality rates, more targeted methods may help to further decrease morbidity and mortality. There are a number of promising new therapies under development for the treatment of IA. A novel echinocandin, Biafungin (CD101), is currently under investigation in pre-clinical studies and showed early promise in mouse models as a once-weekly dosing option for the prevention and treatment of some IFIs, including IA [92]. Other agents are under investigation in clinical trials, some with novel mechanisms of action. E1210 (Eisai Company, Tokyo, Japan) is investigating a novel agent that targets the glycosylphosphatidylinositol (GP1) protein in the cell wall leading to the arrest of fungal growth. E1210 was promising in pre-clinical studies with in vitro activity against most yeast and molds including *A. fumigatus* and is entering phase I clinical trials [93]. F901318 (FTG Ltd., Cheshire, United Kingdom) targets pyrimidine biosynthesis and has shown in vitro activity against triazole-resistant molds such as *A. fumigatus*, *Scedosporium* spp., and *Fusarium* spp. in Phase I study [94]. Phase II studies are currently ongoing. ASP2397 (Vical Inc., San Diego, CA, USA) is a novel agent that inhibits aluminum iron chelation and has shown promise in Phase I clinical trials against *Aspergillus* spp. [95]. 

### 3.2. Treatment of Chronic Pulmonary Aspergillosis

In the only randomized-controlled trial of azole therapy (*n* = 17) versus supportive care (*n* = 14) there was a significant benefit from a six-month course of itraconazole 400 mg daily [96]. Consequently, triazoles including itraconazole and voriconazole are the treatment of choice for CPA, with AII recommendation in the guideline published by the ESCMID, the European Respiratory Society (ERS), and the ECMM and a strong recommendation in guideline published by the IDSA [44,88]. Posaconazole (BII) can be used as an alternative [44].

## 4. Conclusions

Infections caused by *Aspergillus* spp. remain associated with high morbidity and mortality. While mold-active antifungal prophylaxis has led to a decrease of occurrence of IA in those patients most at risk for infection, IA is increasingly observed in other non-neutropenic patient groups, including critically-ill patients with influenza, patients with caspase recruitment domain family member 9 (CARD9) deficiency [97], or patients who receive biological therapies, such as tumor necrosis factor-α inhibitors, and new small molecule kinase inhibitors, such as ibrutinib [98]. Clinical presentations of IA in many of these patient groups are atypical and diagnosis remains challenging. Early and targeted systemic antifungal treatment remains the most important predictive factor for successful outcome in immunocompromised individuals, and recent guidelines recommend both voriconazole and isavuconazole for primary treatment of IA. Few studies evaluated treatment options for CPA, where long-term oral itraconazole or voriconazole remain the treatment of choice.

## Figures and Tables

**Table 1:** Pharmacokinetic Characteristics of Broad-Spectrum Triazoles, Liposomal Amphotericin B, and Echinocandins [48,49,50,51,52].

	Voriconazole	Isavuconazole	Posaconazole	Liposomal Amphotericin B	Caspofungin	Anidulafungin
**Dosage**	IV: 6 mg/kg Q12 × 24 h, 4 mg/kg Q12 starting Day 2 PO: 200 mg Q12 h	IV: 200 mg Q8 h × 48 h, 200 mg daily starting Day 3 PO: 200 mg Q8 h for 48 h, 200 mg daily starting Day 3	IV: 300 mg Q12 × 24 h, 300 mg daily starting Day 2 Delayed-release: 300 mg Q12 ×24 h, 300 mg daily starting Day 2 Oral suspension: 200 mg TID	IV: 3 mg/kg/day	70 mg daily on Day 1, 50 mg daily starting Day 2	200 mg daily on Day 1, 100 mg starting Day 2
**Formulation**	PO, IV	PO, IV	PO, IV	IV	IV	IV
**Half-Life (h)**	6	110–115	27–35	7–10	9–11	24–26
**Bioavailability**	Oral, 96%	Oral, 98%	Tablet: 54%Oral suspension: Variable	Oral, 9%	Oral, <5%	Oral, <5%
**Linear PK**	No	High	IV: NoSuspension/Tablet: High	Yes, doses 1–3 mg/kg; No at higher doses	Yes (Animal Model)	Yes (Animal Model)
**Renal Excretion**	2%	<1%	<1%	4.5%	41%	<1%
**CNS Penetration**	High	High (Animal Model)	Low	High (Animal Model)	Low (Animal Model)	Low (Animal Model)
**Metabolism**	CYP2C19, CYP2C9, CYP3A4	CYP3A4/5	UGT	Unknown	Hydrolysis and *N*-acetylation	Spontaneous biotransformation into inactive peptide

**Table 2:** Recommendations for the Treatment of Invasive Pulmonary Aspergillosis.

**Targeted Treatment of Invasive Pulmonary Aspergillosis in Hematologic Malignancy**					
	Voriconazole	Isavuconazole	Itraconazole	Liposomal Amphotericin B	Echinocandins
**ECIL-6 (61)**	AI	AI	CIII	BI	CII
**ESCMID (60)**	AI-AII	AI-AII	CII-CIII	BII	CII-CIII
**IDSA (83)**	AI	AII	-	AII	AII (not recommended)
**Salvage Treatment for Invasive Pulmonary Aspergillosis in Hematologic Malignancy**					
	Voriconazole	Isavuconazole	Itraconazole	Liposomal Amphotericin B	Posaconazole
**ECIL-6 (61)**	BII	BII	CIII	BII	BII
**ESCMID (60)**	AII	AII	CII-DIII	BII	BII
**IDSA (83)**	-	-	AII	AII	AII
**Combination Treatment for Invasive Pulmonary Aspergillosis in Hematologic Malignancy**					
	Voriconazole + Echinocandin		Other combinations		
**ECIL-6 (61)**	CI		CIII		
**ESCMID (60)**	CI-CII		DIII		
**IDSA (83)**	CII		CII		
**Targeted Treatment of Chronic Pulmonary Aspergillosis**					
	Voriconazole	Isavuconazole	Itraconazole	Liposomal Amphotericin B	Posaconazole
**ECIL-6 (61)**	-	-	-	-	-
**ESCMID (60)**	AII	-	AII	-	BII
**IDSA (83)**	-	-	Preferred	-	-

## Abbreviations

AI	strong recommendation, high-quality evidence (IDSA); strong evidence and evidence from ≥1 RCT (ECIL-6); strong recommendation and evidence from ≥1 RCT (ESCMID)
AII	strong recommendation, moderate-quality evidence (IDSA); strong evidence and evidence from ≥1 non-randomized clinical trial, cohort, or case-controlled study (ECIL-6); strong recommendation and evidence from ≥1 non-randomized clinical trial, cohort, or case-controlled study (ESCMID)
AIII	strong recommendation, low-quality evidence (IDSA); strong evidence and opinion from respected authorities (ECIL-6); strong recommendation and opinion of respected authorities (ESCMID)
BI	strong or moderate evidence and evidence from ≥1 RCT (ECIL-6); moderate support and evidence from ≥1 RCT (ESCMID)
BII	strong or moderate evidence and evidence from ≥1 non-randomized clinical trial, cohort, or case-controlled study (ECIL-6); moderate support and evidence from ≥1 non-randomized clinical trial, cohort, or case-controlled study (ESCMID)
CI	weak recommendation, high-quality evidence (IDSA); insufficient evidence for efficacy and evidence from ≥1 RCT (ECIL-6); marginal support and evidence from ≥1 RCT (ESCMID)
CII	weak recommendation, moderate-quality evidence (IDSA); insufficient evidence for efficacy and evidence from ≥1 non-randomized clinical trial, cohort, or case-controlled study (ECIL-6); marginal support and evidence from ≥1 non-randomized clinical trial, cohort, or case-controlled study (ESCMID)
CIII	weak evidence, low-quality evidence (IDSA); insufficient evidence for efficacy and opinion from respected authorities (ECIL-6); marginal support and evidence of respected authorities (ESCMID)
DIII	moderate evidence against efficacy and opinion from respected authorities (ECIL-6); recommendation against use and evidence of respected authorities (ESCMID)

## References

[1] Alastruey-Izquierdo A., Cadranel J., Flick H., Godet C., Hennequin C., Hoenigl M., Kosmidis C., Lange C., Munteanu O., Page I. (2018). Treatment of chronic pulmonary aspergillosis: Current standards and future perspectives. Respiration.

[2] Denning D.W., Page I.D., Chakaya J., Jabeen K., Jude C.M., Cornet M., Alastruey-Izquierdo A., Bongomin F., Bowyer P., Chakrabarti A. (2018). Case definition of chronic pulmonary aspergillosis in resource-constrained settings. Emerg. Infect. Dis..

[3] Kontoyiannis D.P., Marr K.A., Park B.J., Alexander B.D., Anaissie E.J., Walsh T.J., Ito J., Andes D.R., Baddley J.W., Brown J.M. (2010). Prospective surveillance for invasive fungal infections in hematopoietic stem cell transplant recipients, 2001-2006: Overview of the transplant-associated infection surveillance network (TRANSNET) Database. Clin. Infect. Dis..

[4] Cornely O.A., Lass-Florl C., Lagrou K., Arsic-Arsenijevic V., Hoenigl M. (2017). Improving outcome of fungal diseases-Guiding experts and patients towards excellence. Mycoses.

[5] Geiser D. (2009). Sexual structures in *Aspergillus*: Morphology, importance and genomics. Med. Mycol..

[6] Bongomin F., Gago S., Oladele R.O., Denning D.W. (2017). Global and multi-national prevalence of fungal diseases-estimate precision. J. Fungi.

[7] Rees J.R., Pinner R.W., Hajjeh R.A., Brandt M.E., Reingold A.L. (1998). The epidemiological features of invasive mycotic infections in the San Francisco Bay area, 1992–1993: Results of population-based laboratory active surveillance. Clin. Infect. Dis..

[8] Pappas P.G., Alexander B.D., Andes D.R., Hadley S., Kauffman C.A., Freifeld A., Anaissie E.J., Brumble L.M., Herwaldt L., Ito J. (2010). Invasive fungal infections among organ transplant recipients: Results of the transplant-associated infection surveillance network (TRANSNET). Clin. Infect. Dis..

[9] Prattes J., Flick H., Pruller F., Koidl C., Raggam R.B., Palfner M., Eigl S., Buzina W., Zollner-Schwetz I., Thornton C.R. (2014). Novel tests for diagnosis of invasive aspergillosis in patients with underlying respiratory diseases. Am. J. Respir. Crit. Care Med..

[10] Bergeron A., Porcher R., Sulahian A., de Bazelaire C., Chagnon K., Raffoux E., Vekhoff A., Cornet M., Isnard F., Brethon B. (2012). The strategy for the diagnosis of invasive pulmonary aspergillosis should depend on both the underlying condition and the leukocyte count of patients with hematologic malignancies. Blood.

[11] Petraitiene R., Petraitis V., Bacher J.D., Finkelman M.A., Walsh T.J. (2015). Effects of host response and antifungal therapy on serum and BAL levels of galactomannan and (1,3)-β-d-glucan in experimental invasive pulmonary aspergillosis. Med. Mycol..

[12] Cornillet A., Camus C., Nimubona S., Gandemer V., Tattevin P., Belleguic C., Chevrier S., Meunier C., Lebert C., Aupée M. (2006). Comparison of epidemiological, clinical, and biological features of invasive aspergillosis in neutropenic and nonneutropenic patients: A 6-year survey. Clin. Infect. Dis..

[13] Blot S.I., Taccone F.S., Van den Abeele A.M., Bulpa P., Meersseman W., Brusselaers N., Dimopoulos G., Paiva J.A., Misset B., Rello J. (2012). A clinical algorithm to diagnose invasive pulmonary aspergillosis in critically ill patients. Am. J. Respir Crit. Care. Med..

[14] Prattes J., Hoenigl M., Krause R., Buzina W., Valentin T., Reischies F., Koidl C., Zollner-Schwetz I. (2017). Invasive aspergillosis in patients with underlying liver cirrhosis: A prospective cohort study. Med. Mycol..

[15] Lewis R.E., Cahyame-Zuniga L., Leventakos K., Chamilos G., Ben-Ami R., Tamboli P., Tarrand J., Bodey G.P., Luna M., Kontoyiannis D.P. (2013). Epidemiology and sites of involvement of invasive fungal infections in patients with haematological malignancies: A 20-year autopsy study. Mycoses.

[16] Hoenigl M., Salzer H.J., Raggam R.B., Valentin T., Rohn A., Woelfler A., Seeber K., Linkesch W., Krause R. (2012). Impact of galactomannan testing on the prevalence of invasive aspergillosis in patients with hematological malignancies. Med. Mycol..

[17] Willinger B., Lackner M., Lass-Florl C., Prattes J., Posch V., Selitsch B., Eschertzhuber S., Hönigl K., Koidl C., Sereinigg M. (2014). Bronchoalveolar lavage lateral-flow device test for invasive pulmonary aspergillosis in solid organ transplant patients: A semiprospective multicenter study. Transplantation.

[18] Pruller F., Wagner J., Raggam R.B., Hoenigl M., Kessler H.H., Truschnig-Wilders M., Krause R. (2014). Automation of serum (1,3)-β-d-glucan testing allows reliable and rapid discrimination of patients with and without candidemia. Med. Mycol..

[19] Prattes J., Lackner M., Eigl S., Reischies F., Raggam R.B., Koidl C., Flick H., Wurm R., Palfner M., Wölfler A. (2015). Diagnostic accuracy of the *Aspergillus*-specific bronchoalveolar lavage lateral-flow assay in haematological malignancy patients. Mycoses.

[20] Eigl S., Prattes J., Reinwald M., Thornton C.R., Reischies F., Spiess B., Neumeister P., Zollner-Schwetz I., Raggam R.B., Flick H. (2015). Influence of mould-active antifungal treatment on the performance of the *Aspergillus*-specific bronchoalveolar lavage fluid lateral-flow device test. Int. J. Antimicrob. Agents.

[21] Prattes J., Hoenigl M., Rabensteiner J., Raggam R.B., Prueller F., Zollner-Schwetz I., Valentin T., Hönigl K., Fruhwald S., Krause R. (2014). Serum 1,3-β-d-glucan for antifungal treatment stratification at the intensive care unit and the influence of surgery. Mycoses.

[22] Eigl S., Prattes J., Lackner M., Willinger B., Spiess B., Reinwald M., Selitsch B., Meilinger M., Neumeister P., Reischies F. (2015). Multicenter evaluation of a lateral-flow device test for diagnosing invasive pulmonary aspergillosis in ICU patients. Crit. Care.

[23] Eigl S., Hoenigl M., Spiess B., Heldt S., Prattes J., Neumeister P., Wolfler A., Rabensteiner J., Prueller F., Krause R. (2017). Galactomannan testing and *Aspergillus* PCR in same-day bronchoalveolar lavage and blood samples for diagnosis of invasive aspergillosis. Med. Mycol..

[24] Heldt S., Hoenigl M. (2017). Lateral flow assays for the diagnosis of invasive aspergillosis: Current status. Curr. Fungal Infect. Rep..

[25] Hoenigl M., Perez-Santiago J., Nakazawa M., de Oliveira M.F., Zhang Y., Finkelman M.A., Letendre S., Smith D., Gianella S. (2016). (1,3)-β-d-glucan: A biomarker for microbial translocation in individuals with acute or early HIV Infection?. Front. Immunol..

[26] Prattes J., Schneditz D., Pruller F., Jaindl E., Sauseng N., Hoenigl M., Schilcher G., Krause R. (2017). 1,3-ss-d-glucan testing is highly specific in patients undergoing dialysis treatment. J. Infect..

[27] Prattes J., Hoenigl M., Zinke S.E., Heldt S., Eigl S., Johnson G.L., Bustin S., Stelzl E., Kessler H.H. (2018). Evaluation of the new AspID polymerase chain reaction assay for detection of *Aspergillus* species: A. pilot study. Mycoses.

[28] Reischies F.M., Prattes J., Pruller F., Eigl S., List A., Wolfler A., Buzina w., Zollner-Schwetz I., Valentin T., Rabensteiner J. (2016). Prognostic potential of 1,3-β-d-glucan levels in bronchoalveolar lavage fluid samples. J. Infect..

[29] Reischies F.M., Raggam R.B., Prattes J., Krause R., Eigl S., List A., Quehenberger F., Strenger V., Wölfler A., Hoenigl M. (2016). Urine galactomannan-to-creatinine ratio for detection of invasive aspergillosis in patients with hematological malignancies. J. Clin. Microbiol..

[30] Orasch T., Prattes J., Faserl K., Eigl S., Duttmann W., Lindner H., Haas H., Hoenigl M. (2017). Bronchoalveolar lavage triacetylfusarinine C (TAFC) determination for diagnosis of invasive pulmonary aspergillosis in patients with hematological malignancies. J. Infect..

[31] McCarthy M.W., Petraitiene R., Walsh T.J. (2017). Nucleic acid amplification methodologies for the detection of pulmonary mold infections. Expert Rev. Mol. Diagn..

[32] Heldt S., Prattes J., Eigl S., Spiess B., Flick H., Rabensteiner J., Johnson G., Prüller F., Wölfler A., Niedrist T. (2018). Diagnosis of invasive aspergillosis in hematological malignancy patients: Performance of cytokines, Asp LFD, and *Aspergillus* PCR in same day blood and bronchoalveolar lavage samples. J. Infect..

[33] Hoenigl M., Eigl S., Heldt S., Duettmann W., Thornton C., Prattes J. (2018). Clinical evaluation of the newly formatted lateral-flow device for invasive pulmonary aspergillosis. Mycoses.

[34] Cordonnier C., Pautas C., Maury S., Vekhoff A., Farhat H., Suarez F., Dhédin N., Isnard F., Ades L., Kuhnowski F. (2009). Empirical versus preemptive antifungal therapy for high-risk, febrile, neutropenic patients: A randomized, controlled trial. Clin. Infect. Dis..

[35] Fortun J., Martin-Davila P., Gomez Garcia de la Pedrosa E., Silva J.T., Garcia-Rodriguez J., Benito D., Venanzi E., Castaño F., Fernández-Ruiz M., Lazaro F. (2016). Galactomannan in bronchoalveolar lavage fluid for diagnosis of invasive aspergillosis in non-hematological patients. J. Infect..

[36] Maertens J., Maertens V., Theunissen K., Meersseman W., Meersseman P., Meers S., Verbeken E., Verhoef G., Van Eldere J., Lagrou K. (2009). Bronchoalveolar lavage fluid galactomannan for the diagnosis of invasive pulmonary aspergillosis in patients with hematologic diseases. Clin. Infect. Dis..

[37] Hoenigl M., Seeber K., Koidl C., Buzina W., Wolfler A., Duettmann W., Wagner J., Strenger V., Krause R. (2013). Sensitivity of galactomannan enzyme immunoassay for diagnosing breakthrough invasive aspergillosis under antifungal prophylaxis and empirical therapy. Mycoses.

[38] Reinwald M., Hummel M., Kovalevskaya E., Spiess B., Heinz W.J., Vehreschild J.J., Schultheis B., Krause S.W., Claus B., Suedhoff T. (2012). Therapy with antifungals decreases the diagnostic performance of PCR for diagnosing invasive aspergillosis in bronchoalveolar lavage samples of patients with haematological malignancies. J. Antimicrob. Chemother..

[39] Reinwald M., Spiess B., Heinz W.J., Vehreschild J.J., Lass-Florl C., Kiehl M., Schultheis B., Krause S.W., Wolf H., Bertz H. (2012). Diagnosing pulmonary aspergillosis in patients with hematological malignancies: A multicenter prospective evaluation of an *Aspergillus* PCR assay and a galactomannan ELISA in bronchoalveolar lavage samples. Eur. J. Haematol..

[40] Springer J., Lackner M., Nachbaur D., Girschikofsky M., Risslegger B., Mutschlechner W., Fritz J., Heinz W.J., Einsele H., Ullmann A.J. (2016). Prospective multicentre PCR-based *Aspergillus* DNA screening in high-risk patients with and without primary antifungal mould prophylaxis. Clin. Microbiol. Infect..

[41] Boch T., Spiess B., Cornely O.A., Vehreschild J.J., Rath P.M., Steinmann J., Heinz W.J., Hahn J., Krause S.W., Kiehl M.G. (2016). Diagnosis of invasive fungal infections in haematological patients by combined use of galactomannan, 1,3-β-d-glucan, *Aspergillus* PCR, multifungal DNA-microarray, and *Aspergillus* azole resistance PCRs in blood and bronchoalveolar lavage samples: Results of a prospective multicentre study. Clin. Microbiol. Infect..

[42] White P.L., Parr C., Thornton C., Barnes R.A. (2013). Evaluation of real-time PCR, galactomannan enzyme-linked immunosorbent assay (ELISA), and a novel lateral-flow device for diagnosis of invasive aspergillosis. J. Clin. Microbiol..

[43] Hoenigl M., Prattes J., Spiess B., Wagner J., Prueller F., Raggam R.B., Posch V., Duettmann W., Wölfler A. (2014). Performance of galactomannan, β-d-glucan, *Aspergillus* lateral-flow device, conventional culture, and PCR tests with bronchoalveolar lavage fluid for diagnosis of invasive pulmonary aspergillosis. J. Clin. Microbiol..

[44] Denning D.W., Cadranel J., Beigelman-Aubry C., Ader F., Chakrabarti A., Blot S., Ullmann A.J., Dimopoulos G., Lange C. (2016). Chronic pulmonary aspergillosis: Rationale and clinical guidelines for diagnosis and management. Eur. Respir. J..

[45] Page I.D., Richardson M.D., Denning D.W. (2016). Comparison of six *Aspergillus*-specific IgG assays for the diagnosis of chronic pulmonary aspergillosis (CPA). J. Infect..

[46] Reischies F., Hoenigl M. (2014). The role of surgical debridement in different clinical manifestations of invasive aspergillosis. Mycoses.

[47] Hoenigl M., Krause R. (2013). Antifungal therapy of aspergillosis of the central nervous system and *Aspergillus* endophthalmitis. Curr. Pharm. Des..

[48] Ching M.S., Raymond K., Bury R.W., Mashford M.L., Morgan D.J. (1983). Absorption of orally administered amphotericin B. lozenges. Br. J. Clin. Pharmacol..

[49] Felton T., Troke P.F., Hope W.W. (2014). Tissue penetration of antifungal agents. Clin. Microbiol. Rev..

[50] Groll A.H., Gullick B.M., Petraitiene R., Petraitis V., Candelario M., Piscitelli S.C., Walsh T.J. (2001). Compartmental pharmacokinetics of the antifungal echinocandin caspofungin (MK-0991) in rabbits. Antimicrob. Agents Chemother..

[51] Stone N.R., Bicanic T., Salim R., Hope W. (2016). Liposomal amphotericin B (AmBisome((R))): A review of the pharmacokinetics, pharmacodynamics, clinical experience and future directions. Drugs.

[52] Lestner J.M., Groll A.H., Aljayyoussi G., Seibel N.L., Shad A., Gonzalez C., Wood L.V., Jarosinski P.F., Walsh T.J., Hope W.W. (2016). Population pharmacokinetics of liposomal amphotericin B in immunocompromised children. Antimicrob. Agents Chemother..

[53] Lass-Florl C., Kofler G., Kropshofer G., Hermans J., Kreczy A., Dierich M.P., Niederwieser D. (1998). In-Vitro testing of susceptibility to amphotericin B is a reliable predictor of clinical outcome in invasive aspergillosis. J. Antimicrob. Chemother..

[54] Elefanti A., Mouton J.W., Verweij P.E., Zerva L., Meletiadis J. (2014). Susceptibility breakpoints for amphotericin B and *Aspergillus* species in an in vitro pharmacokinetic-pharmacodynamic model simulating free-drug concentrations in human serum. Antimicrob. Agents Chemother..

[55] Reichert-Lima F., Lyra L., Pontes L., Moretti M.L., Pham C.D., Lockhart S.R., Schreiber A.Z. (2018). Surveillance for azoles resistance in *Aspergillus* spp. highlights a high number of amphotericin B-resistant isolates. Mycoses.

[56] Zoran T., Sartori B., Sappl L., Aigner M., Sanchez-Reus F., Rezusta A., Chowdhary A., Taj-Aldeen S.J., Arendrup M.C., Oliveri S. (2018). Azole-Resistance in *Aspergillus terreus* and related species: An emerging problem or a rare phenomenon?. Front. Microbiol..

[57] Ellis M., Spence D., de Pauw B., Meunier F., Marinus A., Collette L., Sylvester R., Meis R., Boogaerts M., Selleslag D. (1998). An EORTC international multicenter randomized trial (EORTC number 19923) comparing two dosages of liposomal amphotericin B for treatment of invasive aspergillosis. Clin. Infect. Dis..

[58] Cornely O.A., Maertens J., Bresnik M., Ebrahimi R., Ullmann A.J., Bouza E., Heussel C.P., Lortholary O., Rieger C., Boehme A. (2007). Liposomal amphotericin B as initial therapy for invasive mold infection: A randomized trial comparing a high-loading dose regimen with standard dosing (AmBiLoad trial). Clin. Infect. Dis..

[59] Cordonnier C., Bresnik M., Ebrahimi R. (2007). Liposomal amphotericin B (AmBisome) efficacy in confirmed invasive aspergillosis and other filamentous fungal infections in immunocompromised hosts: A pooled analysis. Mycoses.

[60] Jenks J., Reed S.L., Seidel D., Koehler P., Cornely O.A., Mehta S.R., Hoenigl M. (2018). Rare Mold Infections Caused by Mucorales, Lomentospora Prolificans and Fusarium, San Diego: The role of antifungal combination therapy. Int. J. Antimicrob. Agents.

[61] Hoenigl M., Prattes J., Neumeister P., Wolfler A., Krause R. (2018). Real-world challenges and unmet needs in the diagnosis and treatment of suspected invasive pulmonary aspergillosis in patients with haematological diseases: An illustrative case study. Mycoses.

[62] Fisher M.C., Hawkins N.J., Sanglard D., Gurr S.J. (2018). Worldwide emergence of resistance to antifungal drugs challenges human health and food security. Science.

[63] Jenks J.D., Salzer H.J., Prattes J., Krause R., Buchheidt D., Hoenigl M. (2018). Spotlight on isavuconazole in the treatment of invasive aspergillosis and mucormycosis: Design, development, and place in therapy. Drug Des. Devel. Ther..

[64] Hoenigl M., Duettmann W., Raggam R.B., Seeber K., Troppan K., Fruhwald S., Prueller F., Wagner J., Valentin T., Zollner-Schwetz I. (2013). Potential factors for inadequate voriconazole plasma concentrations in intensive care unit patients and patients with hematological malignancies. Antimicrob. Agents Chemother..

[65] Ullmann A.J., Aguado J.M., Arikan-Akdagli S., Denning D.W., Groll A.H., Lagrou K., Lass-Flörl C., Lewis R.E., Munoz P., Verweij P.E. (2018). Diagnosis and management of *Aspergillus* diseases: Executive summary of the 2017 ESCMID-ECMM-ERS guideline. Clin. Microbiol. Infect..

[66] Tissot F., Agrawal S., Pagano L., Petrikkos G., Groll A.H., Skiada A., Lass-Flörl C., Calandra T., Viscoli C., Herbrecht R. (2017). ECIL-6 guidelines for the treatment of invasive candidiasis, aspergillosis and mucormycosis in leukemia and hematopoietic stem cell transplant patients. Haematologica.

[67] Hoenigl M., Raggam R.B., Salzer H.J., Valentin T., Valentin A., Zollner-Schwetz I., Strohmeier A.T., Seeber K., Wölfler A., Sill H. (2012). Posaconazole plasma concentrations and invasive mould infections in patients with haematological malignancies. Int. J. Antimicrob. Agents.

[68] Hoenigl M., Duettmann W., Raggam R.B., Huber-Krassnitzer B., Theiler G., Seeber K., Prueller F., Zollner-Schwetz I., Prattes J., Wagner J. (2014). Impact of structured personal on-site patient education on low posaconazole plasma concentrations in patients with haematological malignancies. Int. J. Antimicrob. Agents.

[69] Prattes J., Duettmann W., Hoenigl M. (2016). Posaconazole plasma concentrations on days three to five predict steady-state levels. Antimicrob. Agents Chemother..

[70] Lenczuk D., Zinke-Cerwenka W., Greinix H., Wolfler A., Prattes J., Zollner-Schwetz I., Valentin T., Lin T.C., Meinitzer A., Hoenigl M. (2018). Antifungal prophylaxis with posaconazole delayed-release tablet and oral suspension in a real-life setting: Plasma levels, Efficacy, and tolerability. Antimicrob. Agents Chemother..

[71] Hoenigl M., Gangneux J.P., Segal E., Alanio A., Chakrabarti A., Chen S.C., Govender N., Hagen F., Klimko N., Meis J.F. (2018). Global guidelines and initiatives from the European Confederation of Medical Mycology to improve patient care and research worldwide: New leadership is about working together. Mycoses.

[72] Herbrecht R., Denning D.W., Patterson T.F., Bennett J.E., Greene R.E., Oestmann J.W., Kern w.v., Marr K.A., Ribaud P., Lortholary O. (2002). Voriconazole versus amphotericin B for primary therapy of invasive aspergillosis. N. Engl. J. Med..

[73] Cornely O.A., Mullane K.M., Ostrosky-Zeichner L., Maher R.M., Croos-Dabrera R., Lu Q., Lademacher C., Perfect J.R., Oren I., Schmitt-Hoffmann A.H. (2018). Isavuconazole for treatment of rare invasive fungal diseases. Mycoses.

[74] Marty F.M., Cornely O.A., Mullane K.M., Ostrosky-Zeichner L., Maher R.M., Croos-Dabrera R., Lu Q., Lademacher C., Oren I., Schmitt-Hoffmann A.H. (2018). Isavuconazole for treatment of invasive fungal diseases caused by more than one fungal species. Mycoses.

[75] Perfect J.R., Cornely O.A., Heep M., Ostrosky-Zeichner L., Mullane K.M., Maher R., Croos-Dabrera R., Lademacher C., Engelhardt M., Chen C. (2018). Isavuconazole treatment for rare fungal diseases and for invasive aspergillosis in patients with renal impairment: Challenges and lessons of the VITAL trial. Mycoses.

[76] Maertens J.A., Raad I.I., Marr K.A., Patterson T.F., Kontoyiannis D.P., Cornely O.A., Bow E.J., Rahav G., Neofytos D., Aoun M. (2016). Isavuconazole versus voriconazole for primary treatment of invasive mould disease caused by *Aspergillus* and other filamentous fungi (SECURE): A phase 3, randomised-controlled, non-inferiority trial. Lancet.

[77] Alexander B.D., Perfect J.R., Daly J.S., Restrepo A., Tobon A.M., Patino H., Hardalo C.H., Graybill J. (2008). Posaconazole as salvage therapy in patients with invasive fungal infections after solid organ transplant. Transplantation.

[78] Walsh T.J., Raad I., Patterson T.F., Chandrasekar P., Donowitz G.R., Graybill R., Greene R.E., Hachem R., Hadley S., Herbrecht R. (2007). Treatment of invasive aspergillosis with posaconazole in patients who are refractory to or intolerant of conventional therapy: An externally controlled trial. Clin. Infect. Dis..

[79] Raad I.I., Hanna H.A., Boktour M., Jiang Y., Torres H.A., Afif C., Kontoyiannis D.P., Hachem R.Y. (2008). Novel antifungal agents as salvage therapy for invasive aspergillosis in patients with hematologic malignancies: Posaconazole compared with high-dose lipid formulations of amphotericin B alone or in combination with caspofungin. Leukemia.

[80] Herbrecht R., Maertens J., Baila L., Aoun M., Heinz W., Martino R., Schwartz S., Ullmann A., Meert L., Paesmans M. (2010). Caspofungin first-line therapy for invasive aspergillosis in allogeneic hematopoietic stem cell transplant patients: An European Organisation for Research and Treatment of Cancer study. Bone Marrow Transplant..

[81] Viscoli C., Herbrecht R., Akan H., Baila L., Sonet A., Gallamini A., Giagounidis A., Marchetti O., Martino R., Meert L. (2009). An EORTC Phase II study of caspofungin as first-line therapy of invasive aspergillosis in haematological patients. J. Antimicrob. Chemother..

[82] Hiemenz J.W., Raad I.I., Maertens J.A., Hachem R.Y., Saah A.J., Sable C.A., Chodakewitz J.A., Severino M.E., Saddier P., Berman R.S. (2010). Efficacy of caspofungin as salvage therapy for invasive aspergillosis compared to standard therapy in a historical cohort. Eur. J. Clin. Microbiol. Infect. Dis..

[83] Marr K.A., Schlamm H.T., Herbrecht R., Rottinghaus S.T., Bow E.J., Cornely O.A., Heinz W.J., Jagannatha S., Koh L.P., Kontoyiannis D.P. (2015). Combination antifungal therapy for invasive aspergillosis: A randomized trial. Ann. Intern. Med..

[84] Denning D.W., Marr K.A., Lau W.M., Facklam D.P., Ratanatharathorn V., Becker C., Ullmann A.J., Seibel N.L., Flynn P.M., Van Buriket J.H. (2006). Micafungin (FK463), alone or in combination with other systemic antifungal agents, for the treatment of acute invasive aspergillosis. J. Infect..

[85] Kontoyiannis D.P., Ratanatharathorn V., Young J.A., Raymond J., Laverdiere M., Denning D.W., Patterson T.F., Facklam D., Kovanda L., Arnold L. (2009). Micafungin alone or in combination with other systemic antifungal therapies in hematopoietic stem cell transplant recipients with invasive aspergillosis. Transpl. Infect. Dis..

[86] Maertens J., Glasmacher A., Herbrecht R., Thiebaut A., Cordonnier C., Segal B.H., Killar J., Taylor A., Kartsonis N., Patterson T.F. (2006). Multicenter, noncomparative study of caspofungin in combination with other antifungals as salvage therapy in adults with invasive aspergillosis. Cancer.

[87] Singh N., Limaye A.P., Forrest G., Safdar N., Munoz P., Pursell K., Houston S., Rosso F., Montoya J.G., Patton P. (2006). Combination of voriconazole and caspofungin as primary therapy for invasive aspergillosis in solid organ transplant recipients: A prospective, multicenter, observational study. Transplantation.

[88] Patterson T.F., Thompson G.R., Denning D.W., Fishman J.A., Hadley S., Herbrecht R., Kontoyiannis D.P., Marr K.A., Morrison V.A., Hong Nguyen M. (2016). Practice guidelines for the diagnosis and management of aspergillosis: 2016 update by the Infectious Diseases Society of America. Clin. Infect. Dis..

[89] Perlin D.S., Rautemaa-Richardson R., Alastruey-Izquierdo A. (2017). The global problem of antifungal resistance: Prevalence, mechanisms, and management. Lancet Infect. Dis..

[90] Schauwvlieghe A.F.A.D., De Jonge N., Van Dijk K., Verweij P.E., Bruggemann R.J., Biemond B.J., Bart A., von dem Borne P.A., Verbon A., van der Beek M.T. (2018). The diagnosis and treatment of invasive aspergillosis in Dutch haematology units facing a rapidly increasing prevalence of azole-resistance. A nationwide survey and rationale for the DB-MSG 002 study protocol. Mycoses.

[91] Van de Veerdonk F.L., Kolwijck E., Lestrade P.P., Hodiamont C.J., Rijnders B.J., van Paassen J., Haas P.J., Oliveira Dos Santos C., Kampinga G.A., Bergmans D.C. (2017). Influenza-Associated Aspergillosis in Critically Ill Patients. Am. J. Respir. Crit. Care Med..

[92] Ong V., Hough G., Schlosser M., Bartizal K., Balkovec J.M., James K.D., Krishnan B.R. (2016). Preclinical evaluation of the stability, safety, and efficacy of CD101, a novel echinocandin. Antimicrob. Agents Chemother..

[93] Hata K., Horii T., Miyazaki M., Watanabe N., Okubo M., Sonoda J., Nakamoto K., Tanaka K., Shirotori S., Murai N. (2011). Efficacy of oral E1210, a new broad-spectrum antifungal with a novel mechanism of action, in murine models of Candidiasis, Aspergillosis, and Fusariosis. Antimicrob. Agents Chemother..

[94] McCarthy M.W., Kontoyiannis D., Cornely O.A., Perfect J.R., Walsh T.J. (2017). Novel agents and drug targets to meet the challenges of resistant fungi. J. Infect. Dis..

[95] Arendrup M.C., Jensen R.H., Cuenca-Estrella M. (2015). In vitro activity of ASP2397 against *Aspergillus* isolates with or without acquired azole resistance mechanisms. Antimicrob. Agents Chemother..

[96] Agarwal R., Vishwanath G., Aggarwal A.N., Garg M., Gupta D., Chakrabarti A. (2013). Itraconazole in chronic cavitary pulmonary aspergillosis: A randomised controlled trial and systematic review of literature. Mycoses.

[97] Rieber N., Gazendam R.P., Freeman A.F., Hsu A.P., Collar A.L., Sugui J.A., Drummond R.A., Rongkavilit C., Hoffman K., Henderson C. (2016). Extrapulmonary *Aspergillus* infection in patients with CARD9 deficiency. JCI Insight.

[98] Chamilos G., Lionakis M.S., Kontoyiannis D.P. (2018). Call for Action: Invasive fungal infections associated with ibrutinib and other small molecule kinase inhibitors targeting immune signaling pathways. Clin. Infect. Dis..

